# Impact of rectal cancer multidisciplinary conferences on patient care plans

**DOI:** 10.1111/codi.70150

**Published:** 2025-06-30

**Authors:** Elizabeth A. Clement, Wenjie Lin, Jerry Liu, Manoj Raval, Ahmer Karimuddin, Terry Phang, Anu Ghuman, Carl Brown

**Affiliations:** ^1^ St Paul's Hospital University of British Columbia Vancouver British Columbia Canada; ^2^ Department of Colorectal Surgery Singapore General Hospital Singapore Singapore

**Keywords:** interdisciplinary communication, patient care planning, patient care team, rectal neoplasms, tertiary care centres

## Abstract

**Aim:**

Multidisciplinary conferences (MDCs) are standard of care for rectal cancer, and literature suggests that MDCs result in changes in as many as 50% of treatments plans. The aim of this study was to determine the frequency of changes to treatment plans at MDCs at our local institution. Secondary outcomes included clinician attendance, change in pathology and radiology reports and the association between tumour stage and care plan changes.

**Method:**

Pre‐ and postconference plans were prospectively collected. Care plan changes were defined as either major (intermodality) or minor (intramodality). Changes to radiology reports (T, N, M, extramural venous invasion or mesorectal fascial status) and pathology reports (primary diagnosis, status of mismatch repair or high‐risk features) were tabulated. Associations between stage and conference plan outcome were determined with Fisher's exact test and multinomial logistic regression.

**Results:**

Pre‐ and postconference plans were prospectively recorded for 44 consecutive meetings. A total of 276 patients were reviewed, with 137 being new diagnoses of rectal adenocarcinoma. Radiology reports were changed in 26% (35/137) of patients and pathology reports were changed in 3% (4/137). Major changes to treatment plans occurred in 12% (17/137) and minor changes occurred in 28% (38/137) of cases. Other than an association between Stage 3 cancers and minor changes, no factor was predictive of care plan changes or confirmation.

**Conclusion:**

MDC review resulted in changes to 40% of treatment plans, and no factors predicted confirmation of pretreatment plans. Our study supports the value of comprehensive review of every rectal cancer by MDC.

**What does this paper add to the literature?**

This study affirms the value of rectal cancer multidisciplinary conferences (MDCs) by demonstrating that they change 40% of patient care plans. Radiology reports change 25% of the time and are frequent drivers of plan alterations. The study bolsters MDC review of all rectal cancers as no single factor predicts plan confirmation.

## INTRODUCTION

The optimal treatment for rectal cancer often requires multimodality therapy. Factors including accurate clinical staging, tumour location relative to the anal sphincter and patient preference can influence neoadjuvant treatment [1, 2]. This is especially true as treatment paradigms become more complex; they can be altered on the basis of tumour genetics and include total neoadjuvant therapy and nonoperative management [3, 4, 5, 6]. Multidisciplinary conferences (MDCs) are used to discuss relevant investigations and patient factors to formulate an optimal treatment strategy, which in turn can facilitate coordination of clinical care.

MDC review has been shown to improve compliance with clinical guidelines and can result in changes to care plans up to 50% of the time, nearly half of which are due to reinterpretation of imaging [7, 8, 9, 10, 11]. It may improve completeness of total mesorectal excision; however, there does not appear to be an effect on circumferential resection margin positivity [12, 13]. There is some suggestion that MDC review could have an impact on survival, but current data are limited [14, 15, 16, 17].

While MDC review of rectal cancer patents is considered standard of care, uptake is not universal [18, 19, 20]. At our tertiary academic centre with five colorectal surgeons, MDCs were established in 2014 and MDC review become a mainstay in our treatment paradigm for all patients with rectal cancer. In this study, our primary objective is to evaluate the impact of our local MDC reviews on treatment plans while secondarily quantifying MDC attendance and associations between tumour stage and care plan changes.

## METHOD

### Multidisciplinary conference process and structure

MDC review at our institution takes place weekly via videoconference. All rectal cancer patients planned for surgery at our hospital are reviewed, and surgeons from outside hospitals within the province can selectively submit complex cases. Preconference plans are sent to conference coordinators. Meeting attendees include colorectal surgeons, pathologists, radiologists, radiation oncologists and medical oncologists. New patients are presented by the attending surgeon, and endoscopic, radiological and pathological images are reviewed. Discussion and treatment recommendations ensue. Conference coordinators take meeting minutes.

### Patient selection, ethics and data collection

All patients presented between 11 June 2021 and 22 April 2022 were included. The study was deemed to be quality improvement by the University of British Columbia Research Ethics Board and was thus exempt from review. A REDCap system was used to prospectively capture all data, including conference attendance, patient demographics, preconference clinical staging, preconference plan, conference attendance, investigations reviewed, postconference staging and postconference plans.

### Analysis

Retrospective analysis was performed. Cases were considered ‘primary review’ when the tumour was newly diagnosed and the patient was pretreatment. Primary reviews of rectal adenocarcinomas were subsequently evaluated. Tumours were defined as low (0–5 cm from the anal verge), mid (>5–10 cm) and high (>10 cm) based on MRI or, if not visible on MRI, on endoscopy.

Pre‐ and postconference plans were categorized into one of seven categories: upfront surgery, neoadjuvant therapy, surveillance, additional workup required, palliative treatment, not yet determined and no plan submitted. Not yet determined was reserved for when the presenting surgeon was evaluating more than one reasonable care path for the patient and submitted both as preconference plans. Subcategories of upfront surgery were diversion, pelvic exent, transanal endoscopic microsurgery, low anterior resection and abdominoperineal resection. Subcategories of neoadjuvant therapy were chemotherapy alone, short‐course radiotherapy, long‐course chemoradiotherapy (CRT) and total neoadjuvant therapy (TNT).

*Major* care plan changes were defined by change in treatment modality (e.g. the preconference plan was for upfront surgery and the postconference plan was for neoadjuvant therapy.) *Minor* changes were intramodality changes. Plans were specified if no preconference plan was submitted, or if the preconference plan was not yet determined. Plans were pended if treatment was put on hold to acquire another investigation.

Our tertiary‐care centre serves a large catchment area, and some patients receive neoadjuvant treatment from oncologists who do not attend our MDCs. Thus, on occasion, neoadjuvant treatment would reflect deference to the local treating physicians. In addition, on occasion, multiple treatment options would be considered appropriate; in this case, MDC recommendation was to present these treatment options for the patient to choose.

Changes in radiology reports included any change in T, N or M stage, or a change in the status of either extramural venous invasion (EMVI) or mesorectal fascia. Changes to pathology reports include change in the primary pathological diagnosis, the mismatch repair (MMR) status or the status of a high‐risk feature (margin, depth of invasion, differentiation, presence of lymphovascular invasion, tumour budding). Not all changes in radiology or pathology reports resulted in altered treatment plans.

### Statistics

Continuous variables are reported as mean ± standard deviation. Fisher's exact test was used to determine any association between stage and conference plan outcome. Multinomial logistic regression analysis was used to determine predictive factors for changes to care plans.

### Outcomes

The primary outcome was frequency of major and minor changes at MDC. Secondary outcomes included clinician attendance, type of investigations reviewed and changes in pathology and radiology reports, as well as the association between tumour stage and postconference plan.

## RESULTS

Between 11 June 2021 and 22 April 2022, 44 MDC meetings took place with 100% attendance from pathology, radiology, surgery and medical oncology. Radiation oncology was present 93% (41/44 meetings) of the time.

A total of 276 patients were reviewed; 169 of these cases were primary reviews of newly diagnosed rectal lesions. Clinicopathological details of all lesions are shown in Table 1; 137 of these lesions were rectal adenocarcinomas, the majority of which were MMR proficient.

**TABLE 1:** Clinicopathological characteristics of patients presented at multidisciplinary conference (MDC).

Total number of patients reviewed	276
Primary review	169
Primary review demographics	
Age (years) (SD)	62 (13)
Sex, *n* (%)	
Male	97 (57%)
Female	72 (43%)
Investigation review, *n* (%)	
Endoscopy	59 (43%)
CT	97 (71%)
MRI	128 (93%)
ERUS	3 (2%)
PET	4 (3%)
Pathology review	25 (18%)
Pathology, *n* (%)	
Rectal adenocarcinoma	137 (81%)
pMMR	114 (67%)
dMMR	2 (1%)
Unknown	21 (12%)
Sigmoid adenocarcinoma	5 (3%)
Anal squamous cell carcinoma	12 (7%)
Neuroendocrine tumour	6 (4%)
Sarcoma/leiomyoma	2 (1%)
Retrorectal cyst	3 (2%)
Melanoma	2 (1%)
Paget's disease	2 (1%)
Preconference staging of rectal adenocarcinomas, *n* (%)	
Stage 1	37 (27%)
Stage 2	25 (18%)
Stage 3	70 (51%)
Stage 4	4 (3%)
Not recorded	1 (1%)
Location of the tumour within the rectum (from the anal verge), *n* (%)	
Low (0–5 cm)	54 (39%)
Mid (>5–10 cm)	52 (38%)
High (>10 cm)	27 (20%)
Unknown	4 (3%)

Abbreviations: ERUS, endorectal ultrasound; MMR, mismatch repair (d, deficient; p, proficient); PET, positron emission tomography.

For primary rectal adenocarcinomas, MRI was reviewed in 128 (93%), CT in 97 (71%), endoscopy images in 59 (43%) and pathology in 25 (18%) patients. Tumours were most commonly Stage 3 (70, 51%); however, there were several Stage 1 (37, 27%) and Stage 2 (25, 18%) tumours. Most were low (54, 39%) or mid (52, 38%) rectal tumours.

Treatment plans were confirmed in 59 of 137 (43%) patients and specified in seven (5%) patients (Table 2). Plans were pended in 16 patients (12%), typically for investigations to confirm the absence/presence of metastatic disease or assess the depth of invasion of a lesion.

**TABLE 2:** Pre‐ and postconference plans for primary review of patients with rectal adenocarcinoma.

Preconference		Postconference														
		Upfront surgery						Neoadjuvant therapy						Watchful waiting	Additional workup	Palliative treatment
		Diversion	TEM	LAR	APR	Exenteration	NYD—patient choice	Chemotherapy	Short course	Long course	TNT	NYD—local decision	Nigro protocol			
Upfront surgery	Diversion	1											1*			
	TEM		2		1										2	
	LAR			10**			1		3**					1	2 **	
	APR				2										1	
	Exenteration					1^										
	NYD		3^		1*		1		1*			1*			5*	
Neoadjuvant therapy	Chemotherapy										1					
	Short course			2				1	5**	2**	3	1*				
	Long course			5*	1					13**	11****	3			3	
	TNT										15****	2			1	
	NYD								1*	3*	4*	2*			1	
Watchful waiting				1^										5^		
Additional workup		1														
Palliative treatment																2*
Not yet determined				1					2*		1*	1*			1	
No plan submitted										1	1					

*Note*: Green = confirmed plans. Yellow = minor change within modality. Blue = major change between modalities. Red = pended. Orange = specified. Asterisks (*) represent patients with changed radiology reports, and carets (^) represent patients with changed pathology reports. Asterisks and carets that are red represent when changed radiology/pathology reports resulted in changes in MDC plans.

Abbreviations: APR, abdominoperineal resection; LAR, low anterior resection; NYD, not yet determined; TEM, transanal endoscopic microsurgery; TNT, total neoadjuvant therapy.

Major (intermodality) changes in treatment plans occurred in 17 cases (12%). Of these, eight patients initially planned for neoadjuvant therapy had postconference plans for upfront surgery and six patients planned for upfront surgery were redirected to neoadjuvant therapy. Of note, one patient initially planned for diversion ended up with a postconference plan for the Nigro protocol in the setting of synchronous rectal adenocarcinoma and anal squamous cell carcinoma.

Minor (intramodality) changes occurred in 38 of 137 cases (28%). Except for six cases, all minor changes represented alterations in neoadjuvant therapy; more than a quarter (11/38) were patients whose preconference plans were CRT and postconference plans included TNT.

Change in radiology reports were seen in 35 patients (26%), with 17 of these being the reason for alteration of treatment plans (12%; three pended, two specified, eight minor and four major changes). Most changes (20/35) represented an upstaging in the T, N or M category. Eight represented downstaging and seven represented neither, which were most commonly changes in the status of EMVI.

Change in pathology reports occurred on four occasions (3%), two of which resulted in care plan changes (one major and one minor change). Most often, these were changes in margin status after endoscopic excision of malignant polyps. One notable change included an original pathology report favouring recurrent prostate cancer which was determined to be a rectal adenocarcinoma via pathology review at MDC. However, this did not change the preconference plan of pelvic exenteration.

Changes in care plans occurred for all stages of rectal cancer (Table 3). Fisher's exact test showed a significant difference (*p* = 0.004), and multinomial logistic regression demonstrated that minor changes to care plans were more common for Stage 3 cancers (*p* = 0.037, Table 4). No other factor was associated with confirmation of or change in care plans, including reinterpretation of investigations or tumour height.

**TABLE 3:** Distribution of outcomes from multidisciplinary conference by clinical stage of cancer.

Postconference outcome by stage					
	Confirmed, *n* (%)	Major, *n* (%)	Minor, *n* (%)	Specified, n (%)	Pended, *n* (%)
Stage 1 (*n* = 37)	19 (51)	6 (16)	4 (11)	1 (3)	7 (19)
Stage 2 (*n* = 25)	8 (32)	6 (24)	4 (16)	2 (8)	5 (20)
Stage 3 (*n* = 70)	29 (41)	4 (6)	**29 (41)**	4 (6)	4 (6)
Stage 4 (*n* = 4)	2 (50)	1 (25)	1 (25)	0 (0)	0 (0)
Total	59 (43)	17 (12)	38 (28)	7 (5)	16 (12)

*Note*: Staging information was missing for one patient whose plan was confirmed, and is included in the total. Fisher's exact test showed a significant difference (*p* = 0.004) and logistic regression (supplemental material) demonstrated that Stage 3 tumours are more likely to have minor changes (*p* = 0.037).

**TABLE 4:** Multinomial logistic regression of MDC outcome including stage, pathology change, radiology change and tumour height.

		Relative risk ratio (95% CI)	*p*‐value
Confirmed plans	(base outcome)		
Major change			
	Stage		
	1 (ref)		
	2	1.72 (0.34–8.71)	0.51
	3	0.28 (0.06–1.37)	0.12
	4	0.92 (0.06–13.71)	0.95
	Pathology change	2.99 (0.15–59.90)	0.47
	Radiology change	2.80 (0.68–11.45)	0.15
	Tumor height		
	Low (ref)		
	Mid	0.55 (0.13–2.39)	0.43
	High	1.54 (0.35–6.69)	0.56
Minor change			
	Stage		
	1 (ref)		
	2	2.00 (0.37–10.86)	0.42
	3	**3.86 (1.09–13.74)**	**0.037**
	4	1.79 (0.12–27.30)	0.68
	Pathology change	2.08 (0.10–41.54)	0.63
	Radiology change	1.14 (0.42–3.10)	0.8
	Tumor height		
	Low (ref)		
	Mid	0.89 (0.33–2.37)	0.81
	High	1.14 (0.34–3.82)	0.83

*Note*: No factors predicted major change and only stage 3 cancers predict minor change. Statistical significance – bolded with a *p*‐value of <0.05.

## DISCUSSION

In this study, MDC review of patients with primary rectal cancer resulted in care plan changes in 40% of cases. While many were minor changes, 12% of care plans underwent major change, representing alterations in the primary modality of treatment. Radiology reports were changed 26% of the time; 12% of care plans were changed due to reinterpretation of images. Pathology reports were rarely changed (3%); however, half of these resulted in care plan changes.

The rate of care plan change is consistent with previously published studies, which have demonstrated that MDC plans change in 25%–53% of patients [7, 8, 10]. Francescutti et al., who showed a 53% change in plans with a modified MDC, attribute their high rate to selective case review [8]. In a subsequent study, however, 35% of care plans were changed when all rectal cancer cases were reviewed [7]. Their rates of change were comparable to those in our study; 8% of presented cases had major changes in care plans and 27% had minor changes. Notably, they used a broader definition of ‘minor change’, which encompassed additional investigations [7]. In our study, we considered these plans ‘pended’.

Snelgrove et al. reported that 29% of care plans changed when a MDC was applied to all cases [10]. Their observed rate of care plan changes due to reinterpretation of imaging studies was 12%, which is the same as ours. While MRI is the most reliable local staging tool in rectal cancer, accuracy is imperfect and bears significant clinical relevance [21, 22, 23]. Appropriately protocoled scans, reporting templates and experienced rectal cancer radiologists can all contribute to accuracy and completeness [23, 24]. Further, clinical context and multimodality anatomical review (i.e. CT, MRI and endoscopic images) are likely to contribute to enhanced MRI interpretation at MDC. It should be noted that revised imaging interpretation does not necessarily mean better accuracy as there is no gold standard. However, our institution is the highest‐volume rectal cancer centre in our provincial jurisdiction and all radiologists are subspeciality gastrointestinal radiologists with high‐volume pelvic MRI practice so it is reasonable to presume that most MDC‐enhanced revisions of MRI interpretation are improvements [24].

In congruence with Francescutti et al., our data did not reveal any factor that predicts major change or plan confirmation [8]. This supports the notion that all rectal cancers should be reviewed by MDC. The only significant finding on multinomial regression was that Stage 3 tumours were more likely undergo minor changes in care plan. These intramodality changes often represent the use of TNT, which was topical at the time that our data were collected [4, 5, 6]. Of the 67 patients whose postconference plans were for neoadjuvant therapy, more than half (34) were planned for TNT. Integration of TNT into treatment paradigms is under debate and, as such, the rapid introduction of TNT strategies in patients who met RAPIDO study criteria (i.e. cT4a/b, cN2, EMVI positive, mesorectal fascial involvement, enlarged lateral nodes) was facilitated by weekly MDC discussion and suggests a knowledge translation advantage of this strategy for patient care. Similarly, it is possible that the importance of MDCs will again be supported as more data become available about treatment paradigms for MMR‐deficient tumours [25, 26]. In the context of this study, however, only two patients (one Stage 2 and one Stage 3) were MMR deficient; at this time, immunotherapy is only available in Canada for patients with unresectable or Stage 4 disease, and thus MMR status played a limited role in this cohort.

Limitations of our study include its prospective design with retrospective analysis as well as the fact that it was completed at a single centre. Also, while our MDCs review rectal cancers at all stages of treatment, our study only analysed newly diagnosed rectal cancers prior to their treatment initiation, which can limit the apparent impact of MDC review. Further, this study did not set out to evaluate the quality of our MDC, which, if done, could contribute to the validity of our findings [10, 27]. That said, at present there is no one best method to evaluate MDCs nor is there a gold standard for MDC format [28, 29]. Finally, we don't report compliance with MDC recommendations, which is due to the distributed nature of our healthcare system. Our MDCs primarily review patients who will receive surgical treatment at St Paul's Hospital in Vancouver, and our conferences include medical and radiation oncologists from our local cancer centre. However, many patients live outside Vancouver and receive their neoadjuvant therapy from cancer centres that are not present at our MDCs, and thus postconference plans remain recommendations. In addition, because surgeons working at hospitals other than St Paul's occasionally present patients for review we don't have access to these patients’ charts for further assessment regarding compliance.

While the debate is dwindling, there is some controversy about the overall utility of MDC review, and more evidence is needed to support its use in rectal cancer [20, 30]. Our study is consistent with previously published data, demonstrating care plan changes in 40% of cases reviewed at a MDC, with nearly half of these being due to reinterpretation of existing investigations (imaging or pathology) by local experts. In addition, we show that even early staged tumours will undergo treatment plan changes with MDC review. In all, this contributes to the body of evidence that MDCs are an essential tool in the treatment of all patients with rectal cancer, especially given the evolution of our treatment paradigms.

## AUTHOR CONTRIBUTIONS

E. A. Clement: conceptualization; methodology; data curation; investigation; validation; formal analysis; writing—original draft; writing—review and editing; project administration. W. Lin: conceptualization; methodology; validation; formal analysis; writing—review and editing. J. Liu: conceptualization; methodology; data curation; investigation; project administration. M. Raval: conceptualization; methodology; data curation; investigation; validation; formal analysis; writing—review and editing; supervision. A. Karimuddin: conceptualization; methodology; data curation; investigation; formal analysis; writing—review and editing; validation; supervision. T. Phang: conceptualization; methodology; data curation; investigation; formal analysis; validation; writing—review and editing; supervision. A. Ghuman: conceptualization; methodology; data curation; investigation; validation; formal analysis; writing—review and editing; supervision. C. Brown: conceptualization; methodology; data curation; investigation; validation; formal analysis; supervision; writing—original draft; writing—review and editing.

## FUNDING INFORMATION

This study received no funding.

## CONFLICT OF INTEREST STATEMENT

The authors have no conflicts of interest to declare.

## ETHICS STATEMENT

Quality improvement as per University of British Columbia Research Ethics Board.

## Data Availability

The data that support the findings of this study are available on request from the corresponding author. The data are not publicly available due to privacy or ethical restrictions.
